# GMPSO-VMD Algorithm and Its Application to Rolling Bearing Fault Feature Extraction

**DOI:** 10.3390/s20071946

**Published:** 2020-03-31

**Authors:** Jiakai Ding, Liangpei Huang, Dongming Xiao, Xuejun Li

**Affiliations:** 1 Hunan Provincial Key Laboratory of Health Maintenance for Mechanical Equipment, Hunan University of Science and Technology, Xiangtan 411201, China; jiakai.ding@mail.hnust.edu.cn (J.D.); huanglp413@163.com (L.H.); hnkjdxlxj@163.com (X.L.); 2 School of Mechatronics Engineering, Foshan University, Foshan 528225, China

**Keywords:** GMPSO, VMD, envelope spectrum, parameter optimization, rolling bearing fault feature extraction

## Abstract

The vibration signal of an early rolling bearing is nonstationary and nonlinear, and the fault signal is weak and difficult to extract. To address this problem, this paper proposes a genetic mutation particle swarm optimization variational mode decomposition (GMPSO-VMD) algorithm and applies it to rolling bearing vibration signal fault feature extraction. Firstly, the minimum envelope entropy is used as the objective function of the GMPSO to find the optimal parameter combination of the VMD algorithm. Then, the optimized VMD algorithm is used to decompose the vibration signal of the rolling bearing and several intrinsic mode functions (IMFs) are obtained. The envelope spectrum analysis of GMPSO-VMD decomposed rolling bearing fault signal IMF1 was carried out. Moreover, the feature frequency of the four fault states of the rolling bearing are extracted accurately. Finally, the GMPSO-VMD algorithm is utilized to analyze the simulation signal and rolling bearing fault vibration signal. The effectiveness of the GMPSO-VMD algorithm is verified by comparing it with the fixed parameter VMD (FP-VMD) algorithm, complete ensemble empirical mode decomposition adaptive noise (CEEMDAN) algorithm and empirical mode decomposition (EMD) algorithm.

## 1. Introduction

Rolling bearings are widely used in rotating machinery. They are also widely used in aircraft engines [1,2] and other rotating machinery [3,4,5,6]. However, rolling bearings are very easily damaged, and their faults are usually located in the outer race of the rolling bearing [7], the inner race of the rolling bearing [8] and the roller element of rolling bearing [9,10].

At present, the industrial monitoring of rolling bearings is based on vibration signal time-frequency analysis [11,12,13,14], and the fault vibration signals of various rolling bearing fault states contain their own unique information, which needs to be identified and classified. Doing so positively impacts health monitoring of the rolling bearing. Of course, the process monitoring of rolling bearings is not only based on vibration signals, but also has some new technologies applied, such as acoustic signals [15], acoustic emission signals [16] and current signals [17,18,19]. Frosini [17] proposed a induction machine rolling bearing fault diagnosis technique based on measuring and analyzing vibration signal and current signal. The flux probe of this method can easily be positioned on the machines and adapted to a wide range of power levels. Immovilli [18] also utilized current signal to diagnose mechanical imbalances and bearing faults. The main contribution of this paper is to describe a simple and effective signal processing technique for current signals. In the field of rolling bearing fault vibration signal processing, there are some common methods of signal processing. For example, wavelet packet decomposition (WPT) [20,21], empirical mode decomposition (EMD) [22,23], complete ensemble empirical mode decomposition adaptive noise (CEEMDAN) [24,25] and local mode decomposition (LMD) [26,27], etc. However, choosing the wavelet basis function in advance at the beginning of the decomposition of the WPT plays an important role, as the signal cannot be decomposed adaptively, which will have a certain impact on the decomposition result of the rolling bearing fault vibration signal. EMD, LMD and CEEMDAN can conduct adaptive decomposition according to the characteristics of the signal, but they all have adverse effects such as mode mixing [28,29], etc. Therefore, the variational mode decomposition (VMD) algorithm was proposed by Dragomiretskiy [30]. The simulation or fault vibration signal could be adaptively decomposed into several narrow-band signals of different frequencies by the VMD algorithm, thus avoiding the adverse effects of mode mixing. Li [31] utilized the VMD algorithm to decompose the internal bearing signal of a high-speed locomotive, which contained a large amount of background noise. The experiment made use of this method to decompose the characteristic signal and achieved a better result. A rolling bearing fault diagnosis method was proposed by Zhang [32]. This method made use of the VMD algorithm to decompose the rolling bearing signal, and compared it with the EMD algorithm: the results showed that the VMD algorithm is better than the EMD algorithm for the extraction of rolling bearing fault features.

However, the selection of the VMD parameters combination [K,α] is particularly important. A parameters-adaptive optimization VMD method based on the grasshopper optimization algorithm (GOA) for rotating machinery vibration signal was proposed by Zhang [33], which used a kurtosis index and correlation coefficient as a fitness function for VMD parameters optimization. A test case showed that this method can effectively find the optimal parameter combination [K,α] in the VMD algorithm. Yan [34] proposed a method to optimize VMD parameters based on the cuckoo search algorithm (CSA). The results showed the CSA-VMD can extract fault features effectively and it was compared with other methods to assess its advantages. Xiao [35] used the average instantaneous frequency as an index to select the most important parameter K of the VMD algorithm, which could find the best parameter K of the algorithm quickly and effectively. Ding [36] utilized a GMPSO algorithm to select the optimal parameters combination [K,α] in the VMD algorithm. Compared with PSO algorithm, the GMPSO algorithm could avoid the occurrence of local optimal value.

Researchers have studied rolling bearing fault classification algorithms very closely. Fault classification algorithms include neural networks (NNs) [37,38,39], support vector machines (SVM) [40,41,42] and deep learning [43,44,45,46]. Among them, SVM needs to determine the kernel function in advance, which lead to low fault diagnosis accuracy. Deep learning needs to learn the fault signal, and the learning time is generally longer than that of neural network, which is not applicable for small fault data samples. In order to find out the fault feature frequency of a rolling bearing, the envelope spectrum analysis of the fault signal component is carried out in this paper. Based on the above literature we investigated methods for mode mixing between EMD algorithm and CEEMDAN algorithm. This paper utilized the GMPSO-VMD algorithm to decompose the rolling bearing fault vibration signal, and then combined it with the envelope spectrum to find fault feature frequency. Therefore, this paper proposes a rolling bearing fault feature extraction method based on GMPSO-VMD algorithm.

## 2. Rolling Bearing Fault Feature Extraction Method

### 2.1. VMD Algorithm

The VMD [30] algorithm decomposes the rolling bearing fault vibration signal data set y(t). The analytic signal of the y(t) is as follows:

(1) $$\left[\left(\delta (t)+\frac{j}{\pi t})\times y_{K}(t\right)\right]e^{-j\omega_{K}t}$$

where: t is time, δ(t) is the impact function.

The VMD algorithm searches for the constrained variational optimal solution by constructing a L2 norm model, expressed as:

(2) $$\begin{array}{l} \underset{\left\{y_{K}(t)\right\},\left\{\omega_{K}\right\}}{\min}\left\{\sum_{K}^{}{\left\|\frac{\partial [\delta (t)+\frac{j}{\pi t}]\times y_{K}(t)}{\partial t}e^{-j\omega_{K}t}\right\|}_{2}^{2}\right\}\\ s.t.\;\sum_{K}^{}y_{K}(t)=y(t) \end{array}$$

where: y(t) is the original rolling bearing fault vibration signal, $y_{K}(t)$ is the modal function, $\omega_{K}$ is the central frequency bandwidth, and δ(t) is the instantaneous impulse signal. $\left(\delta (t)+\frac{j}{\pi t})*y_{K}(t\right)$ is the Hilbert transform for y(t).

This L2 norm model (1) introduces the Lagrange equation to solve it. The parameters of penalty factor α and the Lagrange multiplication operator λ(t) are introduced to construct a non-constraint optimization model. The non-constraint optimization model is defined as Equation (3):

(3) $$\begin{array}{ll} L\left(\left\{y_{K}(t)\right\},\left\{\omega_{K}\right\},\left\{\lambda (t)\right\}\right)= & \alpha \sum_{K}^{}{\left\|\frac{\partial [\delta (t)+\frac{j}{\pi t}]\times y_{K}(t)}{\partial t}e^{-j\omega_{K}t}\right\|}_{2}^{2}\\ & +{\left\|y(t)-\sum_{K}^{}y_{K}(t)\right\|}_{2}^{2}+\lambda (t)\times \left(y(t)-\sum_{K}^{}y_{K}(t)\right) \end{array}$$

The alternate direction method of multipliers (ADMM) is utilized to calculate the variation problem. The result of ${\hat{y}}_{K}{}^{n+1}(\omega )$ is calculated by ADMM as shown in Equation (4).

The VMD algorithm comprises the following steps:

Step 1. Perform an iterative loop n=n+1;

Step 2. After iteration, the IMFs $\left\{{\hat{y}}_{K}(\omega )\right\}$ is updated according to the above formula;

(4) y^Kn+1(ω)=y^(ω)−∑i≠Ky^i(ω)+λ^i(ω)21+2α(ω−ωK)2

Step 3. Update the center frequency $\left\{{\hat{\omega }}_{K}\right\}$ according to the above formula;

(5) $${\hat{\omega }}_{K}{}^{n+1}=\frac{\int_{0}^{\infty }\omega {\left|y_{K}(\omega )\right|}^{2}d\omega }{\int_{0}^{\infty }{\left|y_{K}(\omega )\right|}^{2}d\omega }$$

Step 4. Update Lagrange multiplication operator $\hat{\lambda }(\omega )$;

(6) $${\hat{\lambda }}^{n+1}(\omega )={\hat{\lambda }}^{n}(\omega )+\tau (\hat{y}(\omega )-\sum_{K}^{}y_{K}{}^{n+1}(\omega ))$$

Step 5. Repeat steps 1–4 until the inequality is established;

(7) $$\sum_{K}{\left\|{\hat{y}}_{K}{}^{n+1}-{\hat{y}}_{K}{}^{n}\right\|}_{2}^{2}/{\left\|{\hat{y}}_{K}{}^{n}\right\|}_{2}^{2}<\varepsilon$$

Step 6. End;

where: $\hat{y}(\omega ),{\hat{y}}_{K}(\omega ),\hat{\lambda }(\omega )$ represent the Fourier transform of $y(t),y_{K}(t),\lambda (t)$ respectively, and ε represents the discriminant accuracy. 

### 2.2. GMPSO Algorithm

Particle swarm optimization (PSO) has global optimization capabilities and good performance but it is prone to local minima. We introduce the GMPSO algorithm, proposed by Ding [36] who applied it to gearbox fault diagnosis. The GMPSO algorithm can effectively prevent the occurrence of local minima. Therefore, this paper selects GMPSO algorithm as the optimization algorithm for the parameters combination [K,α]. The particle position and velocity update formula of GMPSO algorithm is shown below:

(8) $$\begin{array}{l} V_{i}^{n+1}=wV_{i}^{n}+C_{1}\eta \left(P_{i}{}^{n}-X_{i}^{n}\right)+C_{2}\eta \left(P_{g}{}^{n}-X_{i}^{n}\right)\\ X_{i}^{n+1}=X_{i}^{n}+V_{i}^{n+1} \end{array}$$

where, w is the inertia factor and its value is non-negative. When it is large, the global optimization ability is strong. η represents the random number in the interval [0,1], $C_{1}$ and $C_{2}$ are acceleration constants, $C_{1}$ is the individual learning factor of each particle, and $C_{2}$ is the social learning factor of each particle. Generally $C_{1}=C_{2}\in [0,4]$, and the number of iterations is n. $P_{i}{}^{n}$ represents the individual extremum of the i variable in the n-th dimension. $P_{g}{}^{n}$ represents the global optimal solution of the i variable in the n-th dimension.

The early fault signal of the rolling bearing is decomposed by the GMPSO-VMD algorithm to obtain a number of IMFs, and then the envelope entropy value of each IMF’s component is calculated separately. The larger the envelope entropy value, the more noise the IMFs component contained, and the less sparse the signal of the component. If the obtained IMFs component contains more fault-related periodic impact characteristic signals, the higher the sparsity of the obtained component signals and the lower the envelope entropy value. Thus, the lower the envelope entropy value, the less noise the IMFs component contained. In this paper, we utilize the envelope entropy $E_{E}$ as the fitness function of the GMPSO algorithm and the minimum envelope entropy as the evaluation index to select the optimal parameters combination [K,α] in the VMD algorithm.

The envelope entropy $E_{E}$ is defined as:

(9) $$E_{j}=A(j)/\sum_{j=1}^{N}A(j)$$

(10) $$E_{E}=-\sum_{j=1}^{N}E_{j}\mathrm{lg}E_{j}$$

j=1,2,⋯,N

where, A(j) is the envelope signal of the original signal after Hilbert transformation, and $E_{j}$ is the normalization of A(j). 

### 2.3. The Proposed Algorithm

Taking the minimum envelope entropy as the fitness function of the GMPSO optimization algorithm proposed in this paper, and taking the parameter K as the particle position in the GMPSO algorithm and the parameter α as the particle velocity in the GMPSO algorithm, the following GMPSO optimization algorithm is constructed. The GMPSO-VMD optimization algorithm model [36] is:

(11) $$\{\begin{array}{c} \begin{array}{c} F=\underset{\beta =[K,\alpha ]}{\min}\left\{E_{E}{}^{IMFs}\right\}\\ s.t.\;K=[1,10] \end{array}\\ \alpha =[0,5000] \end{array}$$

where, F represents the GMPSO algorithm fitness function, $E_{E}{}^{IMFs}$ represents the envelope entropy of each IMF after the original signal is decomposed by GMPSO-VMD, β=[K,α] is the optimization parameter combination. In this paper, K represents the number on the interval [1,10], and α represents the number on the interval [0,5000].

**Figure 1:**
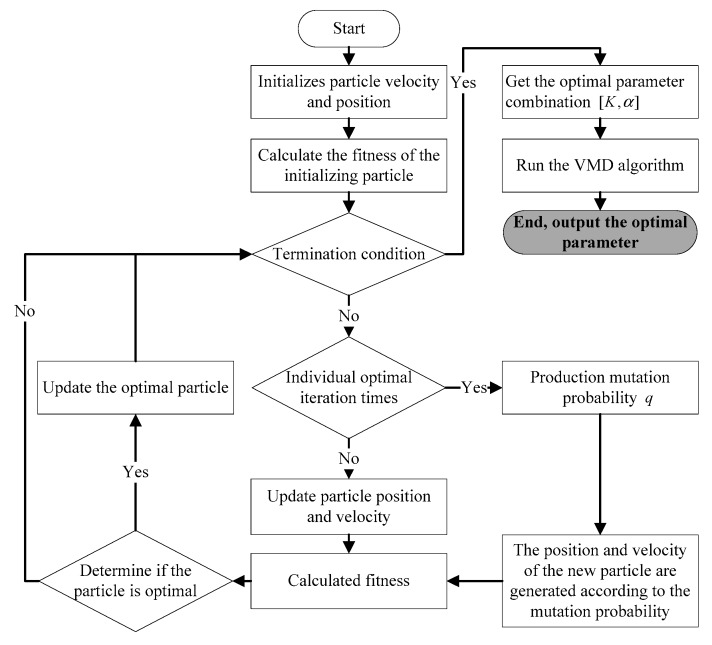
The flow chart of the proposed algorithm in this paper.

Figure 1 shows the flow chart of GMPSO-VMD algorithm.

Steps of the proposed algorithm in this paper are as follows:

(1) Initialize parameters such as particle position and velocity in the GMPSO algorithm.
(2) The particle position and velocity in GMPSO algorithm are taken as the parameter combination [K,α] in the VMD algorithm.
(3) The GMPSO algorithm is implemented to find the optimal combination of the VMD parameter combination [K,α].
(4) The fitness value $\min E_{E}{}^{IMFs}$ is compared so that the local extremum and the global extremum were updated.
(5) When the number of iterations fails to reach the maximum number, the positions of particles reach the local extremum and do not meet the requirements. The GMPSO algorithm will generate the next generation of particle positions and velocities with mutation probability q, so as to avoid the occurrence of the local extremum of PSO algorithm.
(6) When the maximum number of iterations is reached, the iteration stops. Output the optimal parameter combination [K,α] in VMD algorithm.

### 2.4. Fault Feature Extraction Method Based on the GMPSO-VMD Algorithm

**Figure 2:**
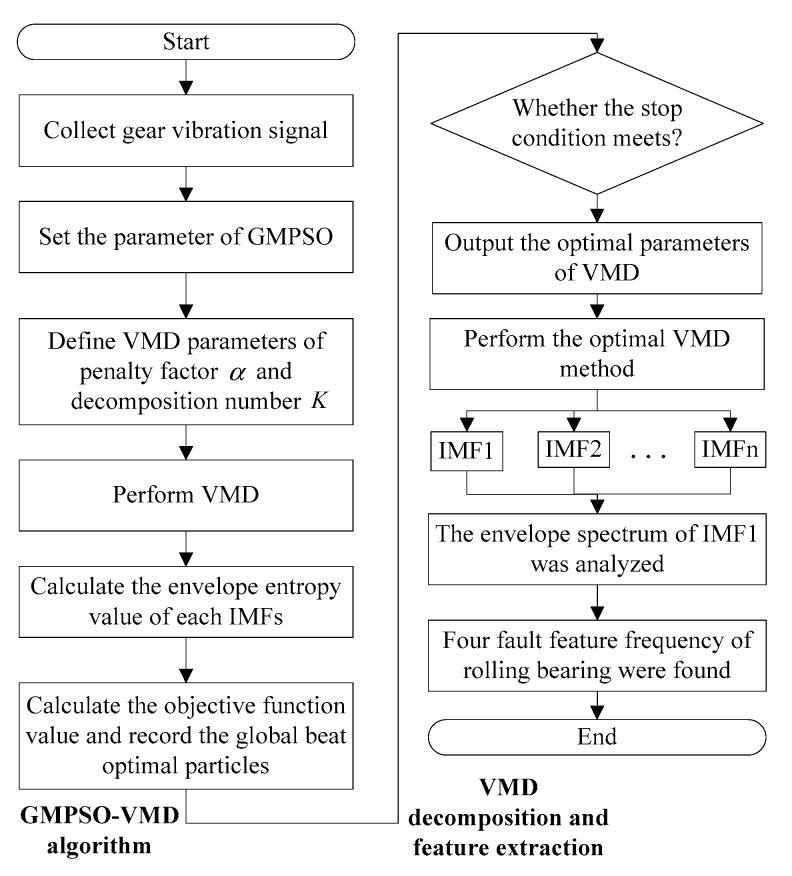
Rolling bearing fault feature extraction flow chart.

In order to effectively extract the feature frequency from the rolling bearing vibration signal, a rolling bearing fault feature extraction method is proposed in this paper. Figure 2 shows a rolling bearing fault feature extraction flow chart.

The following detailed steps are the details of Figure 2:

Step 1. The rolling bearing vibration signal under four working conditions of normal bearing, inner race fault, roller element fault and outer race fault are adopted.

Step 2. The GMPSO algorithm is applied to the parameter combination optimization in the VMD algorithm.

Step 3. The GMPSO-VMD algorithm is utilized for each rolling bearing fault vibration signal.

Step 4. The envelope spectrum of IMF1 is analyzed.

## 3. Simulation Signal Analysis

**Figure 3:**
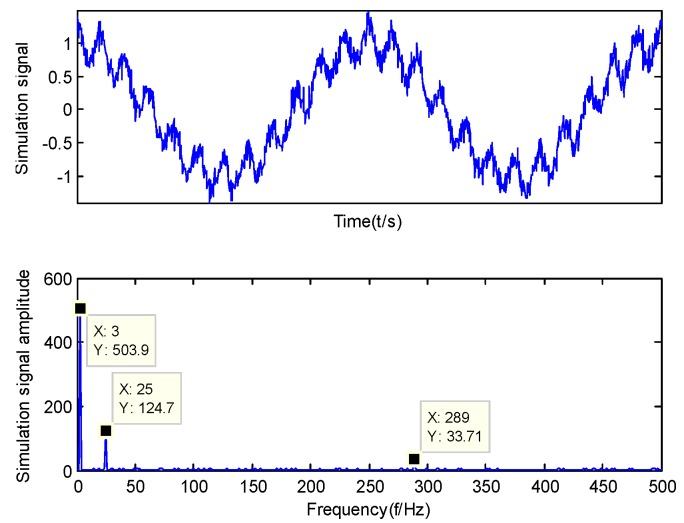
Spectrum diagram and time domain diagram of the y(t).

In order to verify the effectiveness of the GMPSO-VMD algorithm, this paper applies GMPSO-VMD to the decomposition of the simulation signal. The simulation signal adopted in the literature [36] is introduced for analysis. The simulation signal is shown as follows:

(12) $$\{\begin{array}{c} y_{1}(t)=\cos(f_{1}t)\\ y_{2}(t)=\cos(f_{2}t)/4\\ y_{3}(t)=\cos(f_{3}t)/16\\ \begin{array}{l} y(t)=y_{1}(t)+y_{2}(t)+y_{3}(t)+n(t)\\ f_{1}=2\pi w_{1},f_{2}=2\pi w_{2},f_{3}=2\pi w_{3} \end{array} \end{array}$$

where, $w_{1}=3$ is the frequency of $y_{1}$, $w_{2}=25$ is the frequency of $y_{2}$, $w_{3}=289$ is the frequency of $y_{3}$, t is time, $y_{1}(t)$ is the simulation signal with amplitude of 1, $y_{2}(t)$ is the simulation signal with amplitude of 0.25, $y_{3}(t)$ is the simulation signal with amplitude of 0.0625 and n(t) is the noise signal. Figure 3 is the spectrum diagram and time domain diagram of the y(t).

**Figure 4:**
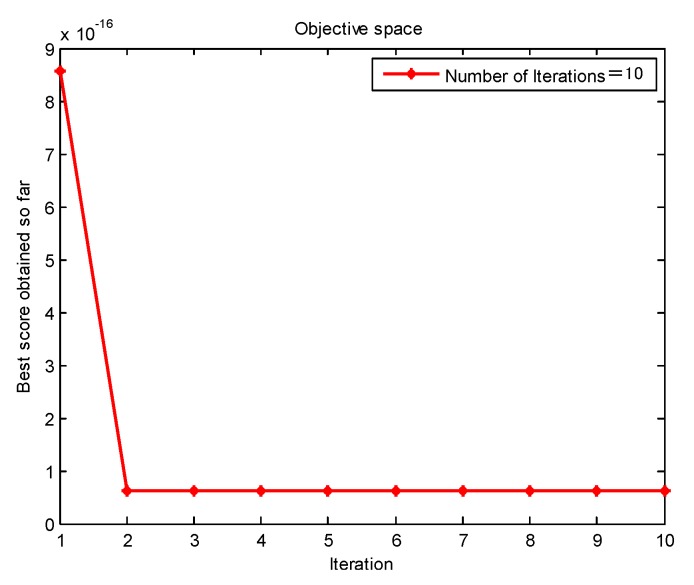
The genetic mutation particle swarm optimization variational mode decomposition (GMPSO) convergence curve of the simulation signal for the variational mode decomposition (VMD) parameter optimization.

Figure 4 shows the change of envelope entropy of the simulation signal with the number of iterations. This paper seeks to find the minimum envelope entropy of the simulation signal. When the envelope entropy reaches the minimum value, the parameter combination [K,α] in the VMD algorithm reaches the optimal value. Further, the optimal parameter combination of [K,α] is obtained as [4,4179] after GMPSO optimization.

**Figure 5:**
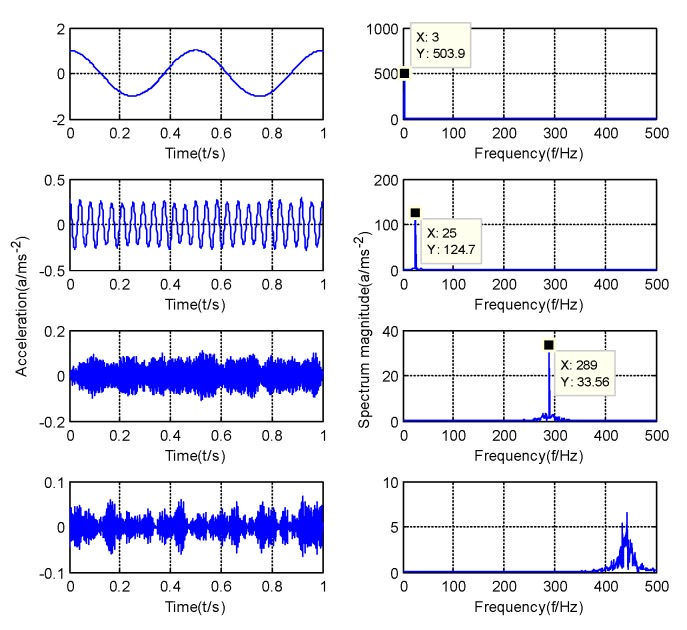
GMPSO-VMD decomposes the simulation signal.

**Figure 6:**
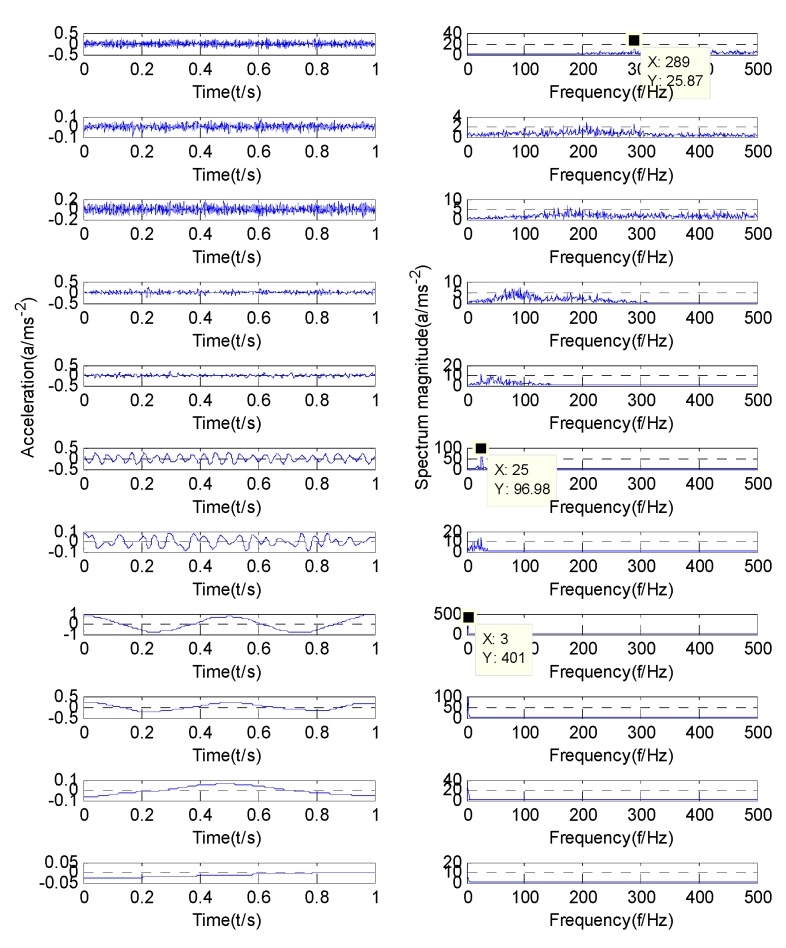
Complete ensemble empirical mode decomposition adaptive noise (CEEMDAN) algorithm decomposes the simulation signal.

According to the optimization results of the GMPSO algorithm, the simulation signal will be decomposed into four components. Figure 5 shows the decomposition results of simulation signal by the GMPSO-VMD algorithm. According to Figure 5, the frequency of IMF1 is $w_{1}=3$, the frequency of IMF2 is $w_{2}=25$, and the frequency of IMF3 is $w_{3}=289$. IMF4 is the noise component. The three feature frequencies are separated accurately by the GMPSO-VMD algorithm. Figure 6 shows the decomposition results of the simulation signal by the CEEMDAN algorithm.

According to Figure 5, the three feature frequencies of the simulation signals are separated correctly. As shown in Figure 6, the frequency of IMF1 is $w_{3}=289$, the frequency of IMF6 is $w_{2}=25$, and the frequency of IMF8 is $w_{1}=3$. The CEEMDAN algorithm separates multiple useless IMFs from the simulation signals, and some of the components have the phenomenon of mode mixing. Among them, mode mixing occurred between IMF6 and IMF7, and the CEEMDAN algorithm separated the simulation signal into several unknown and useless components. Among them, IMF2 to IMF5 are useless components of noise. The signal separation accuracy of the CEEMDAN algorithm is slightly lower than that of the GMPSO-VMD algorithm.

## 4. Experiment Data Analysis

**Figure 7:**
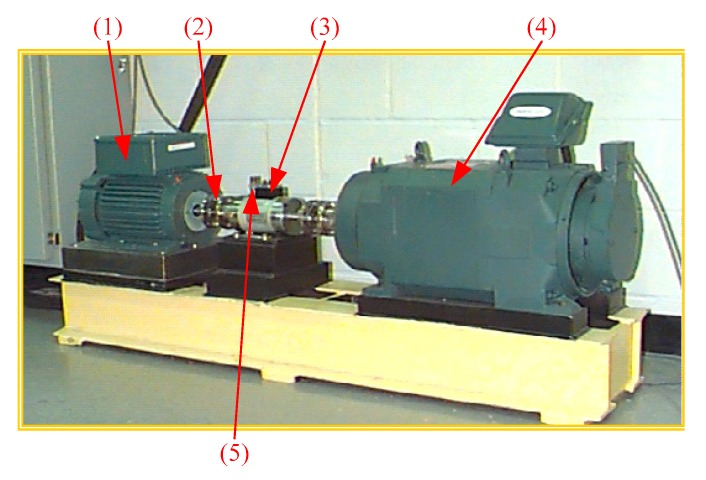
Rolling bearing failure test rig of CWRU: (**1**) three-phase induction motor, (**2**) self-aligning coupling, (**3**) torque sensor, (**4**) fan, and (**5**) acceleration sensor.

**Figure 8:**
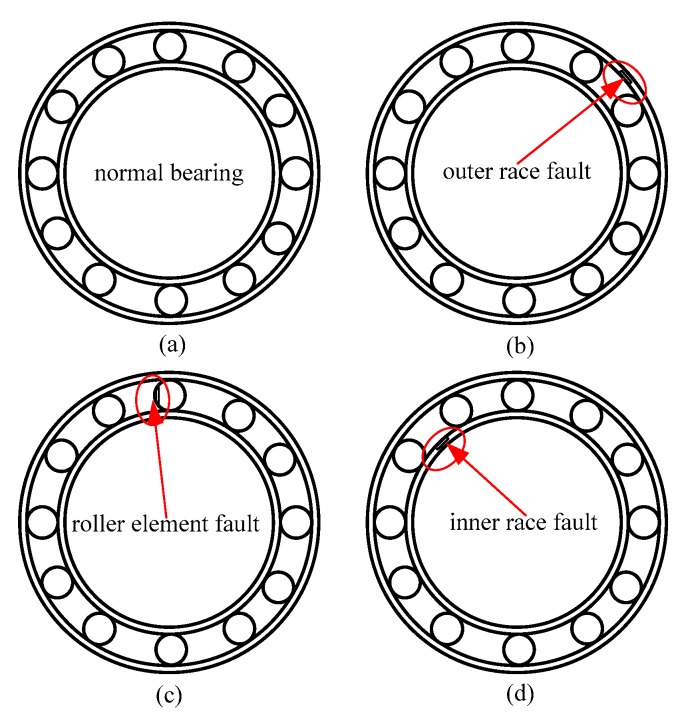
Schematic diagram of a normal rolling bearing and several types of fault: (**a**) normal bearing, (**b**) inner race fault, (**c**) roller element fault, and (**d**) outer race fault.

The rolling bearing data under actual working conditions were applied to the GMPSO-VMD algorithm proposed in this paper to test its effectiveness in extracting fault features of rolling bearings. The rolling bearing fault data set of Case Western Reserve University (United States) (CWRU) [47] was used for experiments and compared with the FP-VMD and EMD algorithms. The rolling bearing test rig of CWRU is shown in Figure 7. In the test rig, the three-phase induction motor (arrow 1 in Figure 7) has a rated power of 1.5 kW, a speed of 1797 r/min, and a current of 3 A. It is connected to a power meter and a torque sensor (arrow 3) through a self-aligning coupling (arrow 2), and drives the fan (arrow 4), where the three-phase induction motor load is 0 kW. At the output end of the three-phase induction motor, the vibration acceleration sensor (arrow 5) is vertically fixed as close as possible to the housing supporting the rolling bearing, and the acceleration sensor (arrow 5) is used for data collection at a sampling frequency of 12 kHz. The rolling bearing model in the test is SKF6205. The inner race, outer race and roller element of the bearing are respectively processed using the electrical discharge machining (EDM) method, which produces tiny pits of 0.117 mm size to simulate the faults of the inner race, outer race and roller elements of the rolling bearing. Table 1. represents the rolling bearing information and notation under an external load of 0 kW. Figure 8 is the schematic diagram of a normal roller bearing, an inner race fault, a roller element fault, and an outer race fault.

In this paper, datasets of rolling bearings under different working conditions of CWRU were adopted for analysis. A total of 32 signal segments of rolling bearings under different working conditions were extracted, 8 signal segments extracted for each working condition.

**Figure 9:**
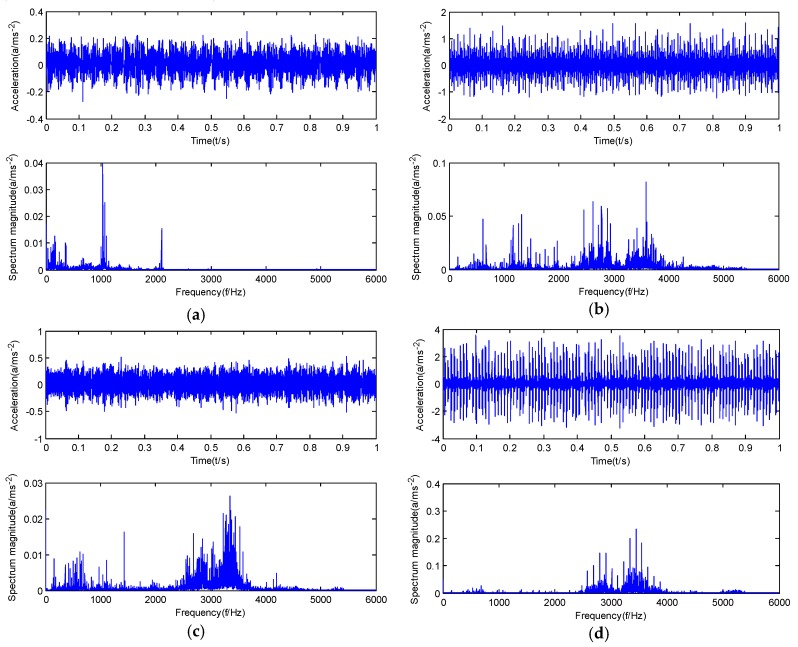
Time domain diagram and spectrum diagram of rolling bearings: (**a**) normal bearing vibration signal, (**b**) inner race fault bearing vibration signal, (**c**) roller element fault bearing vibration signal, and (**d**) outer race fault bearing vibration signal.

The time domain diagrams and spectrum diagrams of vibration signals of normal bearings, inner race faults, roller element faults and outer race faults are shown in Figure 9. As shown in Figure 9, the rolling bearing fault signal features weakly and contains a large number of noise frequencies. Among them, the feature frequency of each fault state is difficult to extract directly from the spectrum diagram, so it needs to be analyzed in the following step. It is necessary to apply the GMPSO-VMD algorithm proposed in this paper to the fault feature extraction of rolling bearing signal under actual working conditions.

**Figure 10:**
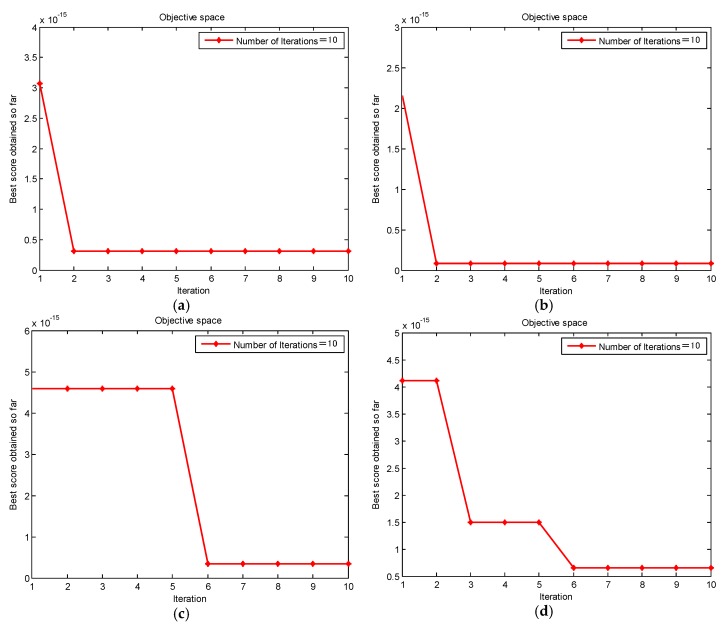
The GMPSO convergence curve for VMD parameter optimization: (**a**) normal bearing vibration signal, (**b**) inner race fault bearing vibration signal, (**c**) roller element fault bearing vibration signal, and (**d**) outer race fault bearing vibration signal.

In this paper, the GMPSO-VMD algorithm is applied to fault feature extraction of the normal bearing, inner race fault, roller element fault and outer race fault, the four types of rolling bearing fault signal. Figure 10a–d shows the GMPSO convergence curve of a normal bearing, an inner race fault of a rolling bearing, a roller element fault of a rolling bearing, and an outer race fault of a rolling bearing vibration signal for VMD parameter optimization.

**Figure 11:**
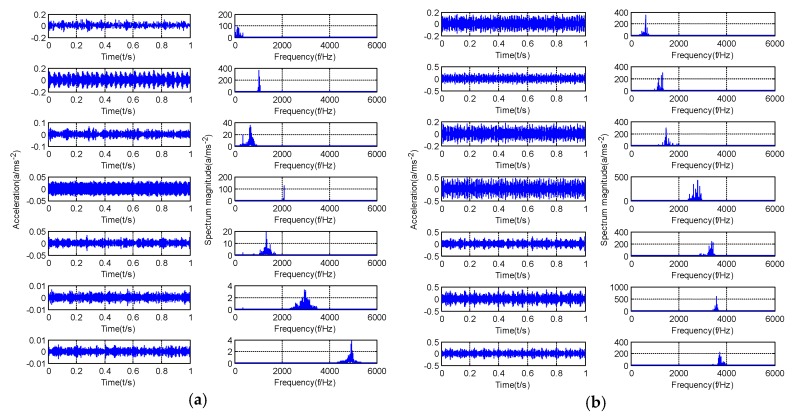

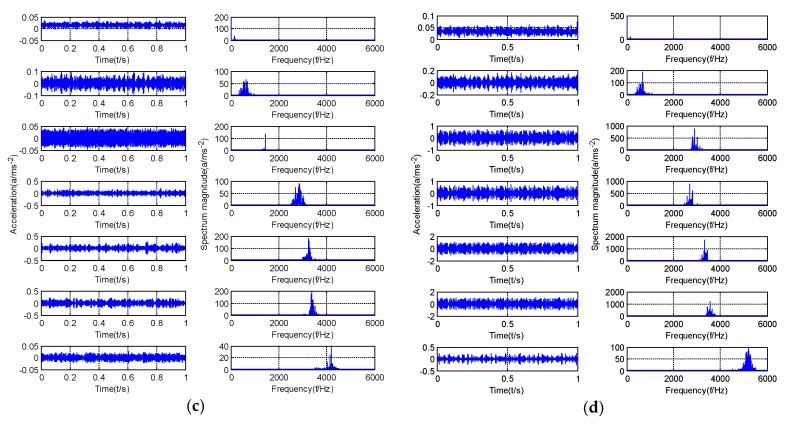
Time domain diagram and spectrum diagram of rolling bearing vibration signal by GMPSO-VMD algorithm decomposition: (**a**) normal bearing, (**b**) inner race fault bearing, (**c**) roller element fault bearing, and (**d**) outer race fault bearing.

The optimal parameter combinations of [K,α] are obtained as [7,2250], [7,3772], [7,4116] and [7,2472] after GMPSO optimization. Figure 11a–d shows the time domain diagram and spectrum diagram of the normal bearing, inner race fault of the rolling bearing, roller element fault of the rolling bearing, outer race fault of the rolling bearing vibration signal decomposed by the GMPSO-VMD algorithm. As shown in Figure 11, the fault signals of rolling bearings were separated successfully, and no mode mixing phenomenon occurred in all IMFs. A large number of high-frequency noise IMFs were obtained.

Some rolling bearing parameters are shown in Table 2. According to the empirical Equations (14)–(16) for the fault frequency of the outer and inner races of the rolling bearing, it can be calculated that the fault frequency of the roller element, outer race and inner race of the rolling bearing are 137.4Hz, 107.3Hz and 162.2Hz. The spindle speed is 1797 r/min so that the motor rotation frequency is 30 Hz.

(13) $$f_{0}=30Hz$$

(14) $$f_{1}=\frac{Z}{2}\times \left(1+\frac{d}{D}\times \cos\alpha \right)\times \frac{N}{60}$$

(15) $$f_{2}=\frac{1}{2}\times \frac{d}{D}\times \left(1-{\left(\frac{d}{D}\right)}^{2}\times \cos^{2}\alpha \right)\times \frac{N}{60}$$

(16) $$f_{3}=\frac{Z}{2}\times \left(1-\frac{d}{D}\times \cos\alpha \right)\times \frac{N}{60}$$

where, z is the number of balls in the rolling bearings; D is the sectional bearing diameter; N is the spindle speed. α is the contact angle of the roller element; d is the diameter of the roll;

The GMPSO-VMD algorithm has a strong mathematical basis, and the frequency of its decomposed IMFs components ranges from small to large. The frequency range of IMF1 is the minimum frequency band. Since the fault frequency of rolling bearings are all between 100 Hz and 200 Hz, this paper selects the IMF1 components decomposed by the GMPSO-VMD algorithm for envelope spectrum analysis. 

However, the essence of the CEEMDAN and EMD algorithm is the iteration of signals, without too much mathematical basis, and both are empirical algorithms. The frequency range of its decomposed IMFs component ranges from large to small, and IMF1 contains the most information. Therefore, the decomposed IMF1 component is used for envelope spectrum analysis.

**Figure 12:**
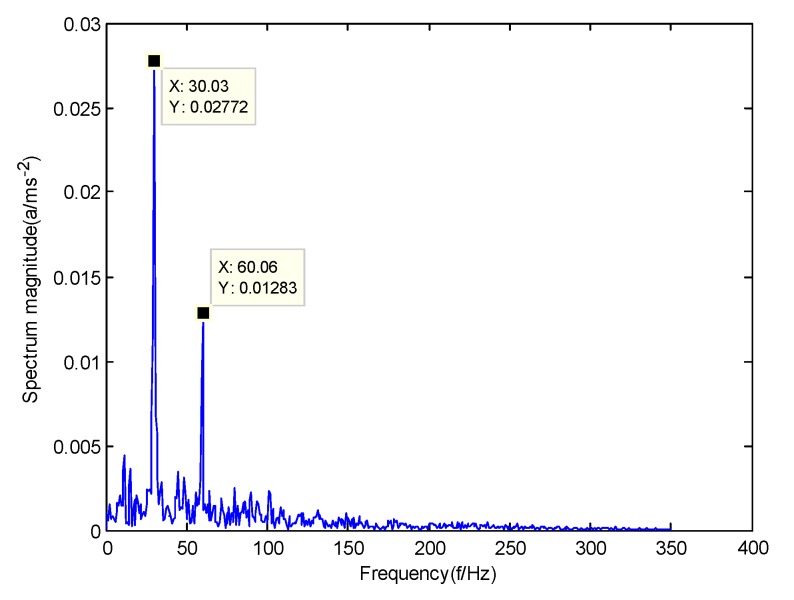
The envelope spectrum of intrinsic mode function 1 (IMF1) of the normal bearing vibration signal obtained using the GMPSO-VMD algorithm.

**Figure 13:**
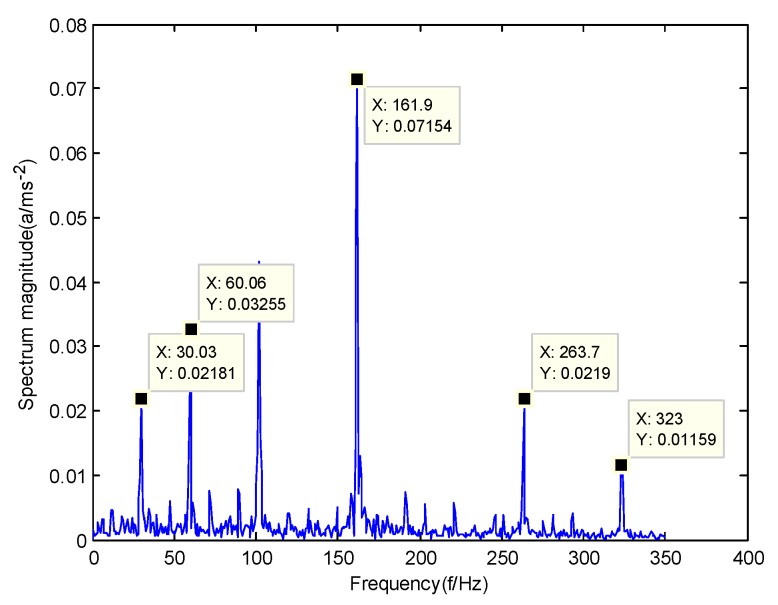
The envelope spectrum of the IMF1 of the inner race fault of the rolling bearing vibration signal obtained using the GMPSO-VMD algorithm.

**Figure 14:**
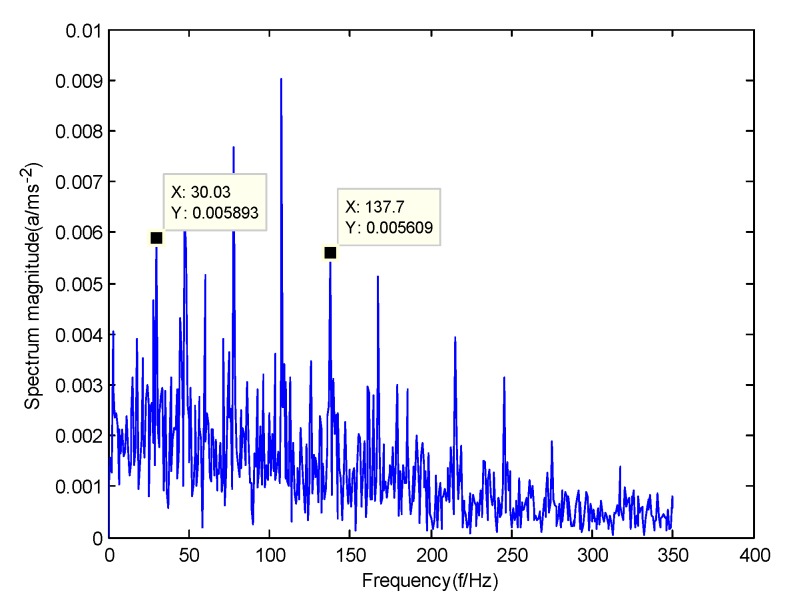
The envelope spectrum of the IMF1 of the roller element fault of the rolling bearing vibration signal obtained using the GMPSO-VMD algorithm.

**Figure 15:**
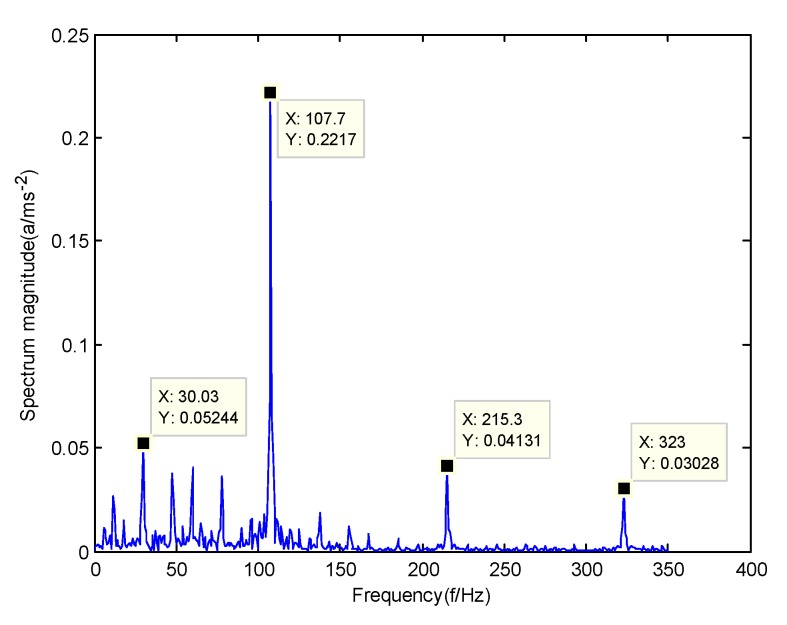
The envelope spectrum of the IMF1 of the outer race fault of the rolling bearing vibration signal obtained using the GMPSO-VMD algorithm.

According to the obtained IMFs after GMPSO-VMD algorithm decomposition, as shown in Figure 11, the spectrum diagram of IMF1 contains the feature frequency of a rolling bearing fault, while the spectrum diagram of other IMFs shows that the fault feature frequency of the rolling bearing is not included. The IMF1 of the fault vibration signal of the four-fault state of rolling bearings are selected as the signal component for envelope spectrum analysis. Figure 12 is the envelope spectrum of the IMF1 of the normal bearing. Figure 13 shows the envelope spectrum of the IMF1 of the inner race fault of the rolling bearing. Figure 14 is the envelope spectrum of the IMF1 of the roller element fault of the rolling bearing. Figure 15 is the envelope spectrum of the IMF1 of the outer race fault of the rolling bearing.

As shown in Figure 12, the motor rotation frequency of the normal bearing is 30.03 Hz, which was obtained accurately from the envelope spectrum of the IMF1 after applying the GMPSO-VMD algorithm. The motor rotation frequency value is close to the theoretical value $f_{0}$. The feature frequency of the normal bearing can be extracted accurately. In addition, the double frequency 60.06 Hz of the motor rotation frequency is also extracted accurately by the envelope spectrum. As shown in Figure 13, the fault frequency of the inner race of the rolling bearing is 161.9 Hz, which was obtained accurately from the envelope spectrum of the IMF1 after use of the GMPSO-VMD algorithm. The inner race fault frequency value is close to the theoretical value $f_{1}$. In addition, the fault feature frequency of the inner race at the double frequency 323 Hz and the motor rotation frequency 30.03 Hz were also extracted accurately by the envelope spectrum. As shown in Figure 14, the fault frequency of the roller element of the rolling bearing was 137.7 Hz, which was obtained accurately from the envelope spectrum of the IMF1 after using the GMPSO-VMD algorithm. The roller element fault frequency value is close to the theoretical value $f_{2}$. In addition, the fault feature frequency of the motor rotation frequency 30.03Hz was also extracted accurately by the envelope spectrum. As shown in Figure 15, the fault frequency of the outer race of the rolling bearing was 107.7Hz, which was obtained accurately from the envelope spectrum of the IMF1 after using the GMPSO-VMD algorithm. The outer race fault frequency value is close to the theoretical value $f_{3}$. In addition, the fault feature frequency of the outer race at the double frequency 215.3 Hz and third harmonic generation 323 Hz, and the motor rotation frequency 30.03 Hz were also extracted accurately by the envelope spectrum.

**Figure 16:**
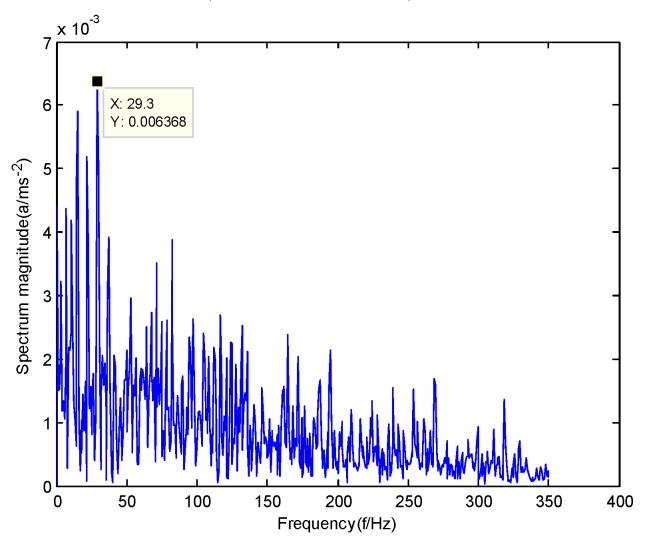
The envelope spectrum of the IMF1 of the normal bearing vibration signal obtained by using the fixed parameter VMD (FP-VMD) algorithm.

**Figure 17:**
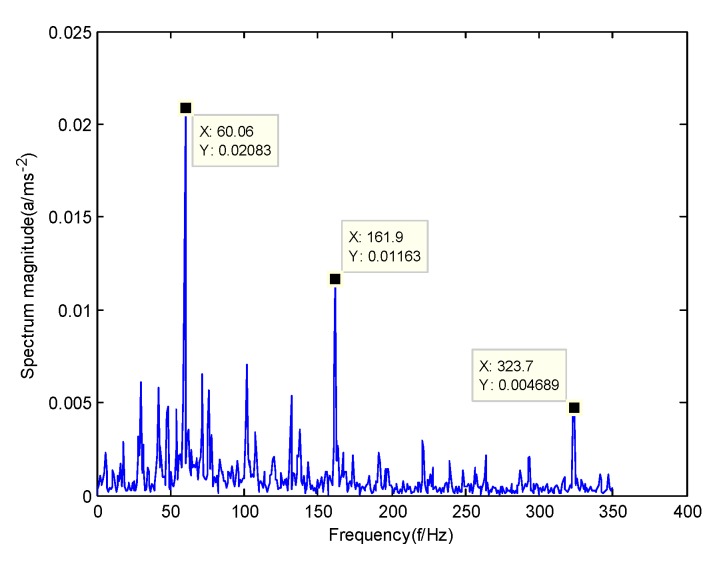
The envelope spectrum of the IMF1 of the inner race fault of the rolling bearing vibration signal obtained by using the FP-VMD algorithm.

**Figure 18:**
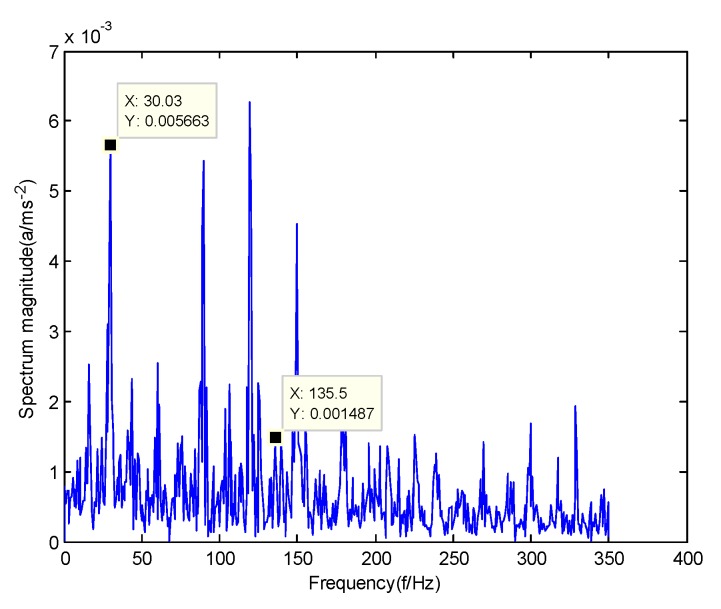
The envelope spectrum of the IMF1 of the roller element fault of the rolling bearing vibration signal obtained by using the FP-VMD algorithm.

**Figure 19:**
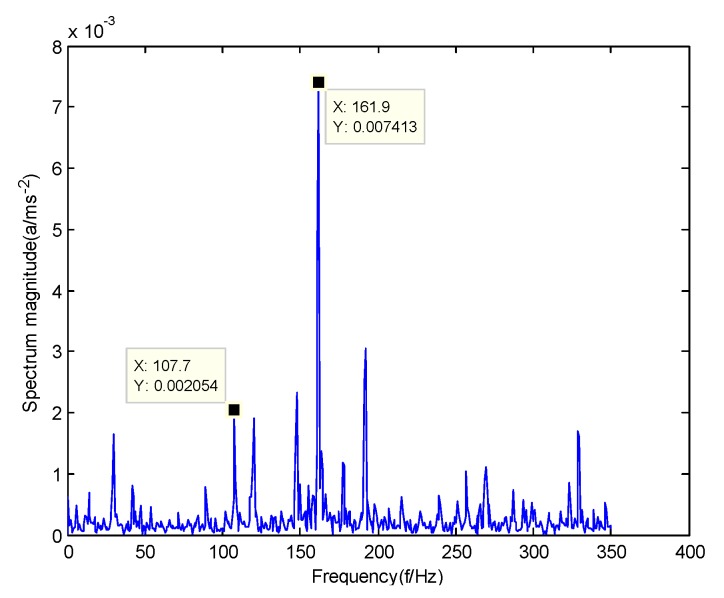
The envelope spectrum of the IMF1 of the outer race fault of the rolling bearing vibration signal obtained by using the FP-VMD algorithm.

In order to verify the difference between the GMPSO-VMD algorithm and other algorithms, the FP-VMD algorithm, CEEMDAN algorithm and EMD algorithm were substituted for the GMPSO-VMD algorithm. Here, the parameters in the FP-VMD algorithm are α=2000 and K=3. Figure 16 is the envelope spectrum of the IMF1 of the normal bearing vibration signal obtained by the FP-VMD algorithm. Figure 17 is the envelope spectrum of the IMF1 of the inner race fault of the rolling bearing vibration signal obtained by the FP-VMD algorithm. Figure 18 is the envelope spectrum of the IMF1 of the roller element fault of the rolling bearing vibration signal obtained by the FP-VMD algorithm. Figure 19 is the envelope spectrum of the IMF1 of the outer race fault of the rolling bearing vibration signal obtained by FP-VMD algorithm.

As shown in Figure 16, the motor rotation frequency of the normal bearing is 30.03 Hz, which was obtained from the envelope spectrum of the IMF1 after use of the FP-VMD algorithm. However, there are many noise frequencies in the envelope spectrum, which lead to inaccurate identification. As shown in Figure 17, the fault frequency of the inner race of the rolling bearing is 161.9 Hz, which was obtained accurately from the envelope spectrum of the IMF1 after using the FP-VMD algorithm. The inner race fault frequency value is close to the theoretical value $f_{1}$. In addition, the fault feature frequency of the inner race at the double frequency of 323.7 Hz and the motor rotation frequency double frequency 60.06 Hz were also extracted accurately by the envelope spectrum. As shown in Figure 18, the fault frequency of the roller element of the rolling bearing is 135.5 Hz, which cannot be obtained accurately from the envelope spectrum of the IMF1 after use of the FP-VMD algorithm. However, unknown frequencies exist in the envelope spectrum, which lead to inaccurate identification. In addition, the fault feature frequency of the motor rotation frequency 30.03 Hz was extracted by the envelope spectrum. As shown in Figure 19, the fault frequency of the outer race of the rolling bearing was 107.7 Hz, which was obtained accurately from the envelope spectrum of the IMF1 after use of the FP-VMD algorithm. In addition, the fault feature frequency of the inner race fault feature frequency, 161.9 Hz, was also extracted accurately by the envelope spectrum. In general, the FP-VMD algorithm was slightly less effective than the GMPSO-VMD algorithm for fault feature extraction of rolling bearing vibration signals.

**Figure 20:**
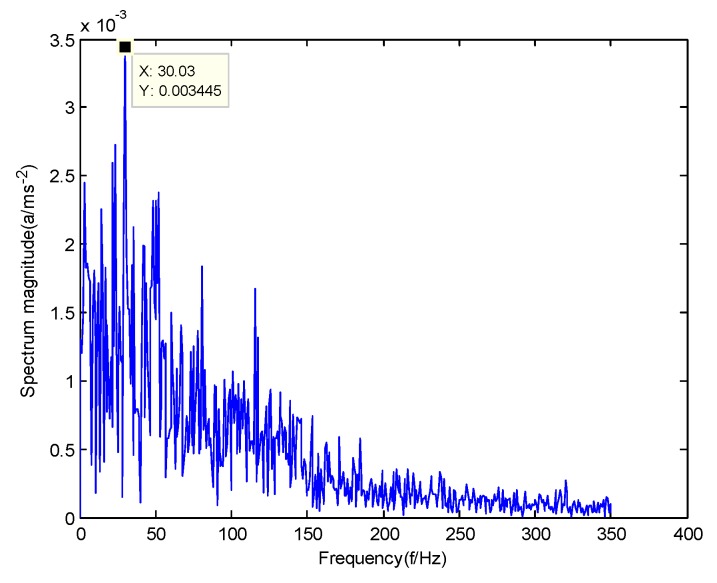
The envelope spectrum of the IMF1 of the normal bearing vibration signal obtained by using the empirical mode decomposition (EMD) algorithm.

**Figure 21:**
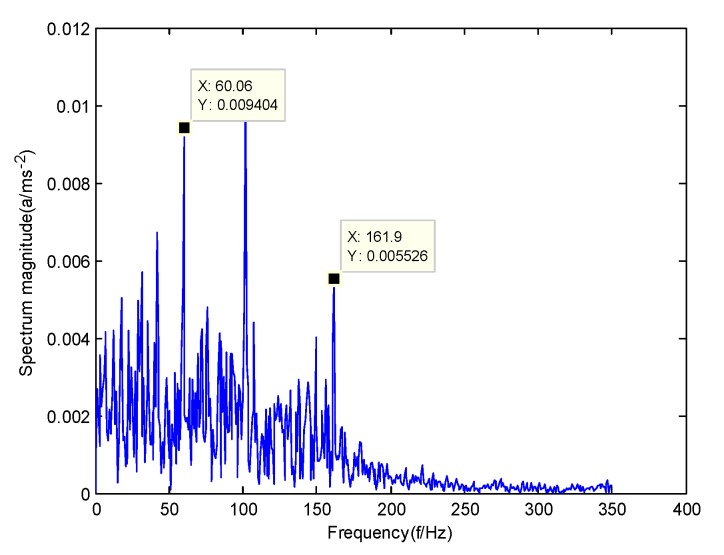
The envelope spectrum of the IMF1 of the inner race fault of the rolling bearing vibration signal obtained by using the EMD algorithm.

**Figure 22:**
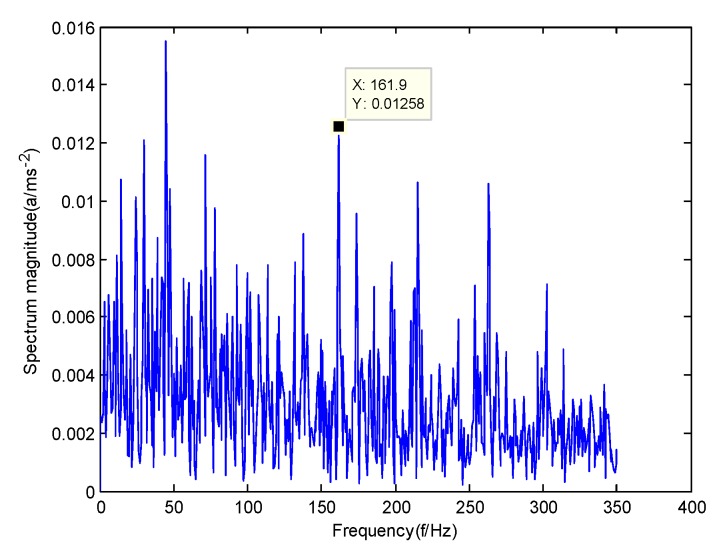
The envelope spectrum of the IMF1 of the roller element fault of the rolling bearing vibration signal obtained by using the EMD algorithm.

**Figure 23:**
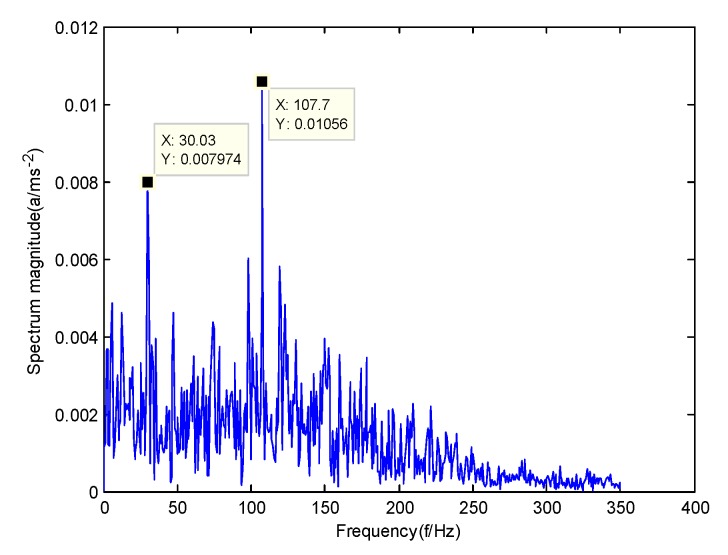
The envelope spectrum of the IMF1 of the outer race fault of the rolling bearing vibration signal obtained by using the EMD algorithm.

Figure 20 is the envelope spectrum of the IMF1 of the normal bearing vibration signal obtained by the EMD algorithm. Figure 21 is the envelope spectrum of the IMF1 of the inner race fault of the rolling bearing vibration signal obtained by the EMD algorithm. Figure 22 is the envelope spectrum of the IMF1 of the roller element fault of the rolling bearing vibration signal obtained by the EMD algorithm. Figure 23 is the envelope spectrum of the IMF1 of the outer race fault of the rolling bearing vibration signal obtained by the EMD algorithm.

As shown in Figure 20, the motor rotation frequency of the normal bearing was 30.03 Hz, which was obtained from the envelope spectrum of the IMF1 using the EMD algorithm. However, there are many noise frequencies in the envelope spectrum, which lead to inaccurate identification. As shown in Figure 21, the fault frequency of the inner race of the rolling bearing was 161.9 Hz, which was obtained from the envelope spectrum of the IMF1 after using the EMD algorithm. However, there are many noise frequencies in the envelope spectrum. In addition, the fault feature frequency of the motor rotation frequency double frequency 60.06 Hz was also extracted accurately by the envelope spectrum. As shown in Figure 22, the fault frequency of the roller element of the rolling bearing was 135.5Hz, which cannot be obtained accurately from the envelope spectrum of the IMF1 after using the EMD algorithm. However, unknown frequencies exist in the envelope spectrum, which lead to inaccurate identification. As shown in Figure 23, the fault frequency of the outer race of the rolling bearing was 107.7 Hz, which was obtained accurately from the envelope spectrum of the IMF1 after using the EMD algorithm. In addition, the fault feature frequency of the motor rotation frequency, 30.03 Hz, was also extracted accurately by the envelope spectrum. In general, the EMD algorithm is less effective than the GMPSO-VMD algorithm for fault feature extraction of rolling bearing vibration signals.

**Figure 24:**
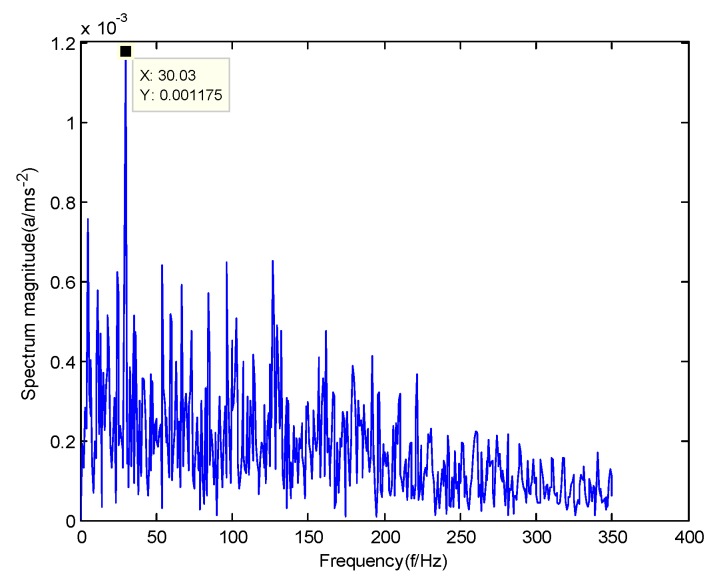
The envelope spectrum of the IMF1 of the normal bearing vibration signal obtained by using the complete ensemble empirical mode decomposition adaptive noise (CEEMDAN) algorithm.

**Figure 25:**
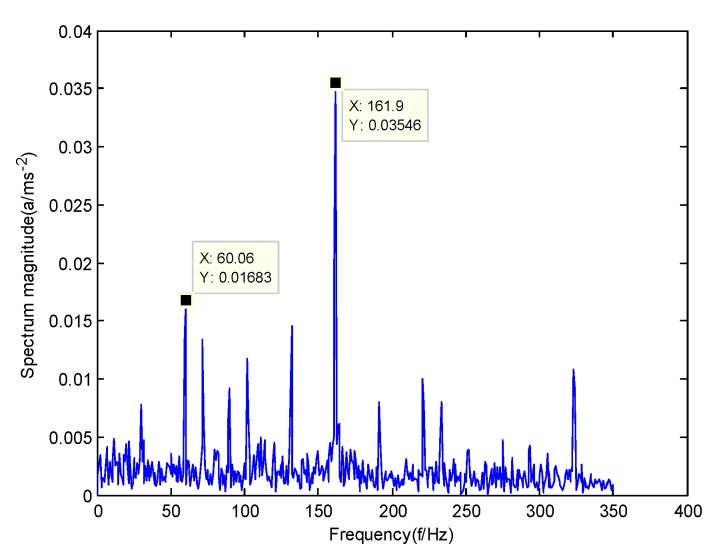
The envelope spectrum of the IMF1 of the inner race fault of the rolling bearing vibration signal obtained by using the CEEMDAN algorithm.

**Figure 26:**
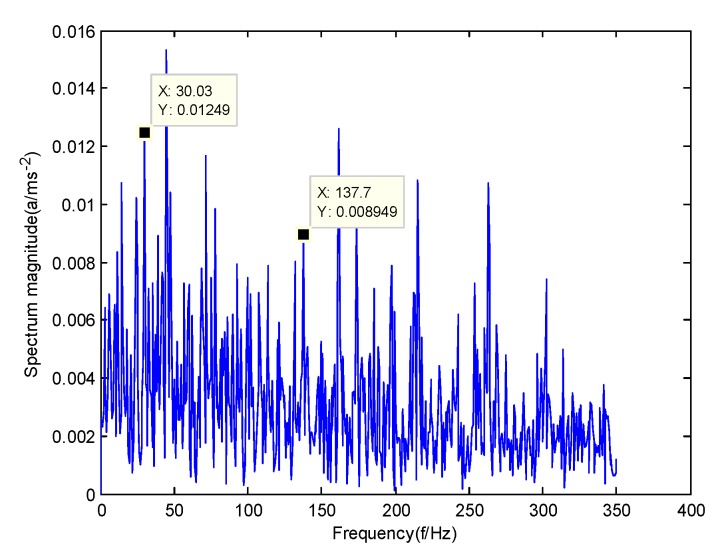
The envelope spectrum of the IMF1 of the roller element fault of the rolling bearing vibration signal obtained by using the CEEMDAN algorithm.

**Figure 27:**
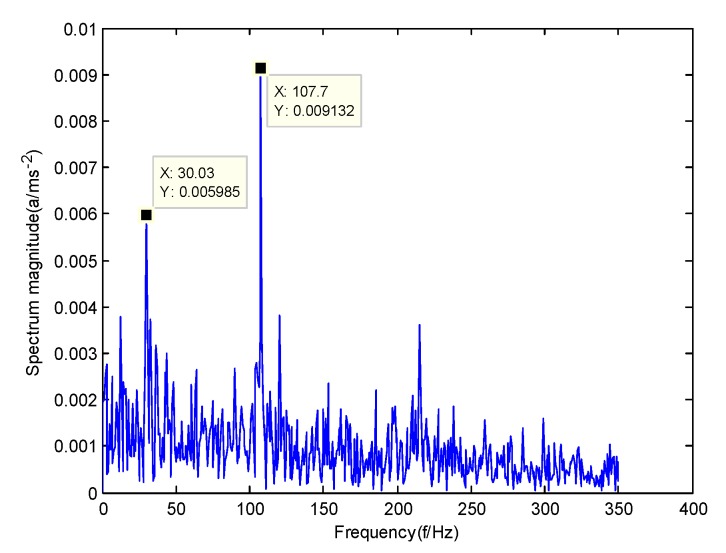
The envelope spectrum of the IMF1 of the outer race fault of the rolling bearing vibration signal obtained by using the CEEMDAN algorithm.

Figure 24 is the envelope spectrum of the IMF1 of the normal bearing vibration signal obtained using the CEEMDAN algorithm. Figure 25 is the envelope spectrum of the IMF1 of the inner race fault of the rolling bearing vibration signal obtained by using the CEEMDAN algorithm. Figure 26 is the envelope spectrum of the IMF1 of the roller element fault of the rolling bearing vibration signal obtained by using the CEEMDAN algorithm. Figure 27 is the envelope spectrum of the IMF1 of the outer race fault of the rolling bearing vibration signal obtained by using the CEEMDAN algorithm.

As shown in Figure 24, the motor rotation frequency of the normal bearing is 30.03 Hz, which was obtained from the envelope spectrum of the IMF1 after using the CEEMDAN algorithm. However, there are many noise frequencies in the envelope spectrum, which lead to inaccurate identification. As shown in Figure 25, the fault frequency of the inner race of the rolling bearing was 161.9 Hz, which was obtained from the envelope spectrum of the IMF1 by using the CEEMDAN algorithm. However, there are many noise frequencies in the envelope spectrum. In addition, the fault feature frequency of the motor rotation frequency double frequency, 60.06 Hz, was also extracted accurately by the envelope spectrum. As shown in Figure 26, the fault frequency of the roller element of the rolling bearing was 137.7Hz, which was obtained from the envelope spectrum of the IMF1 by use of the CEEMDAN algorithm. The roller element fault frequency value was close to the theoretical value $f_{2}$. In addition, the fault feature frequency of the motor rotation frequency, 30.03 Hz, was also extracted by the envelope spectrum. However, there are many noise frequencies in the envelope spectrum, which lead to inaccurate identification. As shown in Figure 27, the fault frequency of the outer race of the rolling bearing was 107.7 Hz, which was obtained from the envelope spectrum of the IMF1 after use of the CEEMDAN algorithm. In addition, the fault feature frequency of the motor rotation frequency, 30.03 Hz, was also extracted accurately by the envelope spectrum. In general, the CEEMDAN algorithm was less effective than the GMPSO-VMD algorithm in fault feature extraction of rolling bearing vibration signals.

The application results show that the GMPSO-VMD algorithm not only has better performance than the FP-VMD, CEEMDAN and EMD algorithms for simulation signals, but also has an advantage over these algorithms for rolling bearing fault feature extraction.

## 5. Conclusions

The GMPSO-VMD algorithm is proposed for analyzing rolling bearing early weak fault vibration signals in this paper. A constraint parameter L2 norm optimization model is established. Due to the influence of mode mixing and fixed parameters, the accuracy of some signal decomposition was reduced, as occurred in the CEEMDAN and FP-VMD algorithms. Furthermore, the effectiveness of the GMPSO-VMD algorithm was confirmed by testing it with both a simulation signal and an experimental rolling bearing signal using actual real-world data. The results show that the GMPSO-VMD algorithm can accurately extract the feature frequency in the form of a spectrum diagram. The main contributions of the GMPSO-VMD algorithm are as follows:

(1) The minimum value of the envelope entropy is taken as the objective function of the GMPSO algorithm to obtain the optimal parameter combination [K,α] of the VMD algorithm.
(2) The accuracy of signal decomposition can be increased by transforming the signal decomposition problem into the parameter optimization problem in the VMD algorithm.
(3) GMPSO-VMD can effectively extract the rotation frequency and fault feature frequency of a rolling bearing vibration signal. Additionally, GMPSO-VMD can accurately classify each type of rolling bearing fault.

The proposed method was verified by using a rolling bearing fault vibration signal. However, the GMPSO-VMD algorithm is still has some problems, such as taking longer to execute than the FP-VMD algorithm. Therefore, we aim to research fast-optimization methods for VMD in the future. Finally, we note that the GMPSO-VMD algorithm can be applied to other fault diagnosis fields, such as fault signal processing applications in welding and additive manufacturing processes.

## Figures and Tables

**Table 1:** Parameters of the rolling bearing data set from CWRU.

Load (kW)	Speed (r/min)	Condition of Rolling Bearing	Fault Diameter (mm)	Notation
0	1797	Normal bearing (N)	/	N
		Inner race fault (IR)	0.117	IR-7
		Roller element fault (RE)	0.117	RE-7
		Outer race fault (OR)	0.117	OR-7

**Table 2:** Rolling bearing parameters.

Roll Diameter (mm)	Section Bearing Diameter (mm)	Contact Angle (°)	Ball Number	Inner Diameter (mm)	Outer Diameter (mm)
7.94	39.04	0	9	25.00	51.97

## Data Availability

The roller bearing vibration data were obtained from the website of Case Western Reverse Laboratory (Cleveland, Ohio, United States) and is available at: https://csegroups.case.edu/bearingdatacenter/pages/download-data-file (accessed 25 October 2015).

## References

[1] Lin T., Chen G., Ouyang W., Zhang Q., Wang H., Chen L. (2018). Hyper-spherical distance discrimination: A novel data description method for aero-engine rolling bearing fault detection. Mech. Syst. Signal Process..

[2] Zhang H., Chen X., Du Z., Li X., Yan R. (2016). Nonlocal sparse model with adaptive structural clustering for feature extraction of aero-engine bearings. J. Sound Vib..

[3] Lei Y., He Z., Zi Y. (2008). A new approach to intelligent fault diagnosis of rotating machinery. Expert Syst. Appl..

[4] Kateris D., Moshou D., Pantazi X.-E., Gravalos I., Sawalhi N., Loutridis S. (2014). A machine learning approach for the condition monitoring of rotating machinery. J. Mech. Sci. Technol..

[5] Heng A., Zhang S., Tan A.C., Mathew J. (2009). Rotating machinery prognostics: State of the art, challenges and opportunities. Mech. Syst. Signal Process..

[6] Sun R., Yang Z., Chen X., Tian S., Xie Y. (2018). Gear fault diagnosis based on the structured sparsity time-frequency analysis. Mech. Syst. Signal Process..

[7] Cui L., Zhang Y., Zhang F., Zhang J., Lee S. (2016). Vibration response mechanism of faulty outer race rolling element bearings for quantitative analysis. J. Sound Vib..

[8] Stack J.R., Habetler T.G., Harley R.G. (2006). Fault-signature modeling and detection of inner-race bearing faults. IEEE Trans. Ind. Appl..

[9] Prabhakar S., Mohanty A., Sekhar A. (2002). Application of discrete wavelet transform for detection of ball bearing race faults. Tribol. Int..

[10] Nikolaou N., Antoniadis I. (2002). Rolling element bearing fault diagnosis using wavelet packets. NDT E Int..

[11] Dybała J., Zimroz R. (2014). Rolling bearing diagnosing method based on empirical mode decomposition of machine vibration signal. Appl. Acoust..

[12] Yan R., Gao R.X. (2006). Hilbert–Huang transform-based vibration signal analysis for machine health monitoring. IEEE Trans. Instrum. Meas..

[13] McInerny S.A., Dai Y. (2003). Basic vibration signal processing for bearing fault detection. IEEE Trans. Educ..

[14] Grasso M., Chatterton S., Pennacchi P., Colosimo B.M. (2016). A data-driven method to enhance vibration signal decomposition for rolling bearing fault analysis. Mech. Syst. Signal Process..

[15] Tandon N., Choudhury A. (1999). A review of vibration and acoustic measurement methods for the detection of defects in rolling element bearings. Tribol. Int..

[16] Miettinen J., Andersson P. (2000). Acoustic emission of rolling bearings lubricated with contaminated grease. Tribol. Int..

[17] Frosini L., Harlisca C., Szabo L. (2014). Induction machine bearing fault detection by means of statistical processing of the stray flux measurement. IEEE Trans. Ind. Electron..

[18] Immovilli F., Bellini A., Rubini R., Tassoni C. (2010). Diagnosis of bearing faults in induction machines by vibration or current signals: A critical comparison. IEEE Trans. Ind. Appl..

[19] Henao H., Capolino G.-A., Fernandez-Cabanas M., Filippetti F., Bruzzese C., Strangas E., Pusca R., Estima J., Riera-Guasp M., Hedayati-Kia S. (2014). Trends in Fault Diagnosis for Electrical Machines: A Review of Diagnostic Techniques. IEEE Ind. Electron. Mag..

[20] Kankar P.K., Sharma S.C., Harsha S.P. (2011). Fault diagnosis of ball bearings using continuous wavelet transform. Appl. Soft Comput..

[21] Sun Q., Tang Y. (2002). Singularity analysis using continuous wavelet transform for bearing fault diagnosis. Mech. Syst. Signal Process..

[22] Yu X., Dong F., Ding E., Wu S., Fan C. (2017). Rolling bearing fault diagnosis using modified LFDA and EMD with sensitive feature selection. IEEE Access.

[23] Guo T., Deng Z. (2017). An improved EMD method based on the multi-objective optimization and its application to fault feature extraction of rolling bearing. Appl. Acoust..

[24] Lei Y., Liu Z., Ouazri J., Lin J. (2017). A fault diagnosis method of rolling element bearings based on CEEMDAN. Proc. Inst. Mech. Eng. Part C J. Mech. Eng. Sci..

[25] Abdelkader R., Kaddour A., Bendiabdellah A., Derouiche Z. (2018). Rolling bearing fault diagnosis based on an improved denoising method using the complete ensemble empirical mode decomposition and the optimized thresholding operation. IEEE Sens. J..

[26] Tian Y., Ma J., Lu C., Wang Z. (2015). Rolling bearing fault diagnosis under variable conditions using LMD-SVD and extreme learning machine. Mech. Mach. Theory.

[27] Darong H., Lanyan K., Bo M., Ling Z., Guoxi S. (2018). A new incipient fault diagnosis method combining improved RLS and LMD algorithm for rolling bearings with strong background noise. IEEE Access.

[28] Zhang L., Wang Z., Quan L. (2018). Research on weak fault extraction method for alleviating the mode mixing of LMD. Entropy.

[29] Hu X., Peng S., Hwang W.-L. (2011). EMD revisited: A new understanding of the envelope and resolving the mode-mixing problem in AM-FM signals. IEEE Trans. Signal Process..

[30] Dragomiretskiy K., Zosso D. (2013). Variational mode decomposition. IEEE Trans. Signal Process..

[31] Li Z., Chen J., Zi Y., Pan J. (2017). Independence-oriented VMD to identify fault feature for wheel set bearing fault diagnosis of high speed locomotive. Mech. Syst. Signal Process..

[32] Zhang M., Jiang Z., Feng K. (2017). Research on variational mode decomposition in rolling bearings fault diagnosis of the multistage centrifugal pump. Mech. Syst. Signal Process..

[33] Zhang X., Miao Q., Zhang H., Wang L. (2018). A parameter-adaptive VMD method based on grasshopper optimization algorithm to analyze vibration signals from rotating machinery. Mech. Syst. Signal Process..

[34] Yan X., Jia M. (2019). Application of CSA-VMD and optimal scale morphological slice bispectrum in enhancing outer race fault detection of rolling element bearings. Mech. Syst. Signal Process..

[35] Xiao D., Ding J., Li X., Huang L. (2019). Gear Fault Diagnosis Based on Kurtosis Criterion VMD and SOM Neural Network. Appl. Sci..

[36] Ding J., Xiao D., Li X. (2020). Gear Fault Diagnosis Based on Genetic Mutation Particle Swarm Optimization VMD and Probabilistic Neural Network Algorithm. IEEE Access.

[37] Ünal M., Onat M., Demetgul M., Kucuk H. (2014). Fault diagnosis of rolling bearings using a genetic algorithm optimized neural network. Measurement.

[38] Wang H., Chen P. (2011). Intelligent diagnosis method for rolling element bearing faults using possibility theory and neural network. Comput. Ind. Eng..

[39] Liu H., Zhou J., Zheng Y., Jiang W., Zhang Y. (2018). Fault diagnosis of rolling bearings with recurrent neural network-based autoencoders. ISA Trans..

[40] Abbasion S., Rafsanjani A., Farshidianfar A., Irani N. (2007). Rolling element bearings multi-fault classification based on the wavelet denoising and support vector machine. Mech. Syst. Signal Process..

[41] Li Y., Xu M., Wei Y., Huang W. (2016). A new rolling bearing fault diagnosis method based on multiscale permutation entropy and improved support vector machine based binary tree. Measurement.

[42] Zheng J., Pan H., Cheng J. (2017). Rolling bearing fault detection and diagnosis based on composite multiscale fuzzy entropy and ensemble support vector machines. Mech. Syst. Signal Process..

[43] Fuan W., Hongkai J., Haidong S., Wenjing D., Shuaipeng W. (2017). An adaptive deep convolutional neural network for rolling bearing fault diagnosis. Meas. Sci. Technol..

[44] Shao H., Jiang H., Zhang H., Duan W., Liang T., Wu S. (2018). Rolling bearing fault feature learning using improved convolutional deep belief network with compressed sensing. Mech. Syst. Signal Process..

[45] Ren L., Cui J., Sun Y., Cheng X. (2017). Multi-bearing remaining useful life collaborative prediction: A deep learning approach. J. Manuf. Syst..

[46] Gan M., Wang C. (2016). Construction of hierarchical diagnosis network based on deep learning and its application in the fault pattern recognition of rolling element bearings. Mech. Syst. Signal Process..

[47] CWRU (2008). Case Western Reserve University Bearing Date Center Website.

